# Generation and functional characterization of the anti-transferrin receptor single-chain antibody-GAL4 (TfRscFv-GAL4) fusion protein

**DOI:** 10.1186/1472-6750-12-91

**Published:** 2012-11-28

**Authors:** Qing Ye, Heyu Hu, Zhihua Wang, Tong Lu, Zhiquan Hu, Xing Zeng, Shu Zhang, Jing Liu, Ping Lei, Cong-Yi Wang, Zhangqun Ye, Guanxin Shen

**Affiliations:** 1 Department of Pathology, Nanjing Drum Tower Hospital affiliated Nanjing University Medical School, Nanjing, People’s Republic of China; 2 Department of Immunology, Tongji Medical College of Huazhong University of Science and Technology (HUST), Wuhan, People’s Republic of China; 3 Department of Urology, Tongji Hospital affiliated Tongji Medical College of Huazhong University of Science and Technology (HUST), Wuhan, People’s Republic of China; 4 The Biomedical Center, Tongji Hospital affiliated Tongji Medical College of Huazhong University of Science and Technology (HUST), Wuhan, People’s Republic of China

## Abstract

**Background:**

The development of vectors for cell-specific gene delivery is a major goal of gene therapeutic strategies. Transferrin receptor (TfR) is an endocytic receptor and identified as tumor relative specific due to its overexpression on most tumor cells or tissues, and TfR binds and intakes of transferrin-iron complex. We have previously generated an anti-TfR single-chain variable fragments of immunoglobulin (scFv) which were cloned from hybridoma cell line producing antibody against TfR linked with a 20 aa-long linker sequence (G_4_S)_4_. In the present study, the anti-TfR single-chain antibody (TfRscFv) was fused to DNA-binding domain of the yeast transcription factor GAL4. The recombinant fusion protein, designated as TfRscFv-GAL4, is expected to mediate the entry of DNA-protein complex into targeted tumor cells.

**Results:**

Fusion protein TfRscFv-GAL4 was expressed in an E. coli bacterial expression system and was recovered from inclusion bodies with subsequent purification by metal-chelate chromatography. The resulting proteins were predominantly monomeric and, upon refolding, became a soluble biologically active bifunctional protein. In biological assays, the antigen-binding activity of the re-natured protein, TfRscFv-GAL4, was confirmed by specific binding to different cancer cells and tumor tissues. The cell binding rates, as indicated by flow cytometry (FCM) analysis, ranged from 54.11% to 8.23% in seven different human carcinoma cell lines. It showed similar affinity and binding potency as those of parent full-length mouse anti-TfR antibody. The positive binding rates to tumor tissues by tissue microarrays (TMA) assays were 75.32% and 63.25%, but it showed weakly binding with hepatic tissue in 5 cases, and normal tissues such as heart, spleen, adrenal cortex blood vessel and stomach. In addition, the re-natured fusion protein TfRscFv-GAL4 was used in an ELISA with rabbit anti-GAL4 antibody. The GAL4-DNA functional assay through the GAL4 complementary conjugation with the GAL4rec-GFP-pGes plasmid to verify the GLA4 activity and GAL4rec-recognized specificity functions. It also shows the complex, TfRscFv-GAL4-GAL4rec-GFP-pGes, could be taken into endochylema to express the green fluorescent protein (GFP) with 8 to 10-fold transfection efficiency.

**Conclusions:**

Results of our study demonstrated that the biofunctianality of genetically engineered fusion protein, TfRscFv-GAL4, was retained, as the fusion protein could both carry the plasmid of GAL4rec-pGes and bind TfR on tumour cells. This product was able to transfect target cells effectively in an immuno-specific manner, resulting in transient gene expression. This protein that can be applied as an effective therapeutic and diagnostic delivery to the tumor using endogenous membrane transport system with potential widespread utility.

## Background

The development of efficient and non-toxic vectors for cell-specific gene delivery is a major challenge in gene therapeutic research. A significant progress has been made in the construction of non-viral vectors that combine different functions required for gene transfer in an artificial complex, as the potential advantages of such a system include ease of use, cost-effective large-scale manufacture, purity, homogeneity, as well as fewer and more well-defined regulatory issues [1,2]. However, the alternative approaches relying on the activities of natural or recombinant DNA-carrier proteins to achieve uptake and intracellular delivery of plasmid DNA has not been developed.

Transferrin (Tf) plays an important role in the cellular iron uptake. Once Tf binds with transferrin receptor (TfR, CD71) on the cell surface, it is ingested into the endosome under acidic condition. During this process, iron is released and the TfR-apo-Tf complexes then re-circulate into the cell surface. Upon disassociation of TfR, apo-Tf regains the ability of binding to iron again. TfR is a cell membrane-associated glycoprotein involved in the cellular uptake of iron and in the regulation of cell growth [3] and it is preferentially expressed in cells with high potential for proliferation. Therefore, the remarkable and stable TfR expression can be detected in various tumor cells such as hepatoma carcinoma cells and leukemia cells [4-7]. Given its abundance in malignant tissues and its significance in the cellular iron uptake, TfR could therefore be used as a biomarker for tumor cells in addition to its relevance in cancer and its extracellular accessibility. These characteristics also render TfR to be an excellent antigen for antibody-based cancer therapy [8]. Indeed, many TfR-specific antibodies (mAbs) have been developed and employed to kill the malignant cells *in vitro* and *in vivo*[9-11]. Previously, we have successfully developed several antibodies against TfR including the human-mouse chimeric antibody, intracellular antibody and bivalent single chain variable fragment (bsFv) antibody [6,12-14]. All of these antibodies displayed tumor-specific biodistribution with substantial antitumor activity. We also employed a transferrin-polyethylenimine (Tf–PEI) delivery system to carry HIF-1α shRNAs into distant tumors. Our studies demonstrated that TfR-mediated endocytosis could induce HIF-1α silencing and resulted in impaired xenograft growth of melanoma *in vivo*[15]. Together, these studies support the possibility that antibodies specific for TfR could be used as a feasible carrier of genes to target for tumor therapy or diagnosis. However, in comparison with the whole antibody or transferin molecule, the scFv has a smaller size for a better penetration into tumor cells. Indeed, low molecule weight single-stranded antibody can be conjugated with various therapeutic or diagnostic molecules to generate targeting complex. Also, the internalized TfR-complex can facilitate the development of tumor therapy or imaging.

The nuclear protein GAL4 is a positive regulator for galactose-induced gene expressions such as GAL1, GAL2, GAL7, GAL10, and MEL1 [16]. The high-affinity of well-characterized of GAL4 DNA-binding has previously been shown to be retained when placed in the context of a heterologous fusion partner or used to enhance gene delivery through conjugation of ligand and other cationic polymers [1,2,17]. GAL4 possesses high binding affinity for a specific 17-bp oligonucleotide sequence (5'-cggrnnrcynyncnccg-3', GAL4rec) and acts as a nuclear localization signal (NLS) [18]. Most importantly, its specific DNA-binding domain (DBD) can bind to the plasmid containing an anti-tumor gene or a specific target gene for imaging. These properties make it a good candidate for use as a vehicle for gene transfer.

In the present study, we developed a particularly appealing approach for the delivery of genes through the use of recombinant protein-based vehicles which consists of a DNA-binding motif fused to a cell-binding TfR-scFv. The TfRscFv-GAL4 protein has a dual function: specific DNA binding via GAL4-DBD and the GAL4rec of the DNA plasmid, and intracellular delivery of the target DNA by TfR-scFv transport. This TfRscFv-GAL4 fusion-mediated DNA delivery system effectively transduced the pGFP-plasmid and induced GFP expression in mammalian cells. We further explored its feasibility for application of tumor-targeted drug delivery and *in vivo* imaging.

## Results

### Construction of TfRscFv-GAL4pET expression vector, expression, purification and renaturation of TfRscFv-GAL4 fusion protein in E. coli

**Figure 1:**
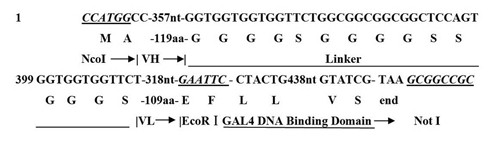
**Construction strategy of TfRscFv-GAL4 prokaryotic expression vector. **The prokaryotic expression vector pET28-a was used as the backbone, the heavy chain (VH) and the light chain (VL) of ScFv was connected with a linker (VH-linker-VL), the His-tag of GAL4 DNA Binding Domain(GAL4-DBD) was fused to the 3’-end of the VH-linker-VL sequence to express the tag conjugated to C-terminal of the single-chain antibody. The whole sequence was cloned into the *Nco*I-*Not*I restriction site to get a prokaryotic expression vector of TfRscFv-GAL4-pET. The steps taken in the construction of each plasmid are detailed in Methods.

The plasmid for TfRscFv-GAL4 fusion protein was constructed as described. Sequencing analysis of the inserts matched exactly with the targeted gene sequences in the database (website: http://www.ncbi.nlm.nh.gov/blast). The full-length sequence for the TfRscFv-GAL4 coding region was identical with the *Nco*I-scFv-*EcoRI*-*GAL4*-*NotI* sequence (Figure 1). 

**Figure 2:**
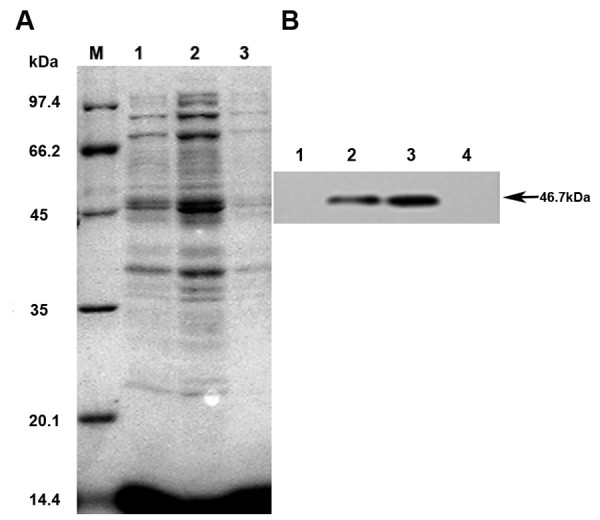
**Expression and purification of TfRscFv-GAL4 fusion protein. A**, SDS-PAGE showing the fusion protein levels present in the inclusion bodies after IPTG induction at different temperature. BL21 *E*.*coli* cell were transformed with TfRscFv-GAL4-pET plasmid and gene expression was showed that Inclusion bodies extract after IPTG induced 24 hours at 37°C (Lane1), 30°C (Lane2), 23°C (Lane3). Lane M: protein molecular weight marker. **B**, after IPTG induced 24 hours at 30°C at different time, Western blotting analysis TfRscFv-GAL4 fusion protein purified from inclusion bodies by IMAC, as showed in 0h (lane 1), 4h (lane 2), 6h (lane 3), and BL_21_*E*.*coli* transformed with an empty vector (lane 4). The highest level of protein expression was at 6h after induction.

High levels TfRscFv-GAL4 of expression of was induced in pET28/TfRscFv-GAL4 transformed BL21 (DE3) E. coli upon the addition of 1mM IPTG. The optimal induction temperature of 30°C was determined to yield the maximum level of protein expression as shown in Lane 2 of Figure 2A. The majority of the fusion protein (~90%) was found not to be soluble but to be contained within the inclusion bodies. Therefore, the TfRscFv-GAL4 containing the His-tagged was purified from the inclusion bodies as described in “Methods.” As shown in Figure 2B, The TfRscFv-GAL4 fusion protein was then purified from cell lysates under denaturing conditions using the Ni–NTA column and re-natured by urea gradient dialysis. SDS-PAGE showed a single band of the re-folded and purified fusion protein with an approximate molecular weight of 45 ~66.2 kDa, in agreement with the expected molecular weight (46.7 kDa) for the recombinant fusion protein, as well as the highest expression were noted 6h after IPTG induction at 30°C. In contrast, no reactive band was detected in BL21 E. coli transformed with an empty vector (Figure 2B). The concentration of re-natured protein was 1.7 mg/ml as measured by the BCA Protein Assay. 

### Immunoreactivity of TfRscFv-GAL4 with various tumor cell lines

**Figure 3:**
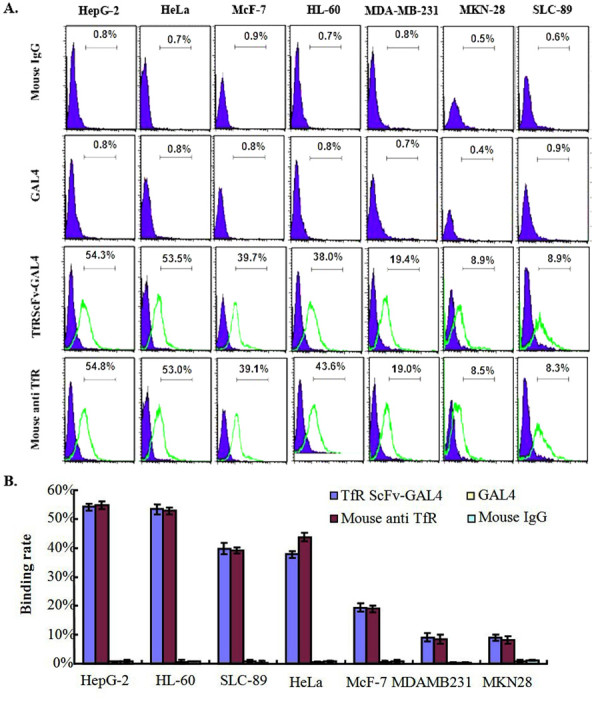
**Flow cytometry demonstrated the immunoreactivity of the TfRscFv-GAL4 to TfR expressed on the surface of different tumor cells. **The cells were incubated with either negative control mouse non-specific IgG, positive control Mouse anti-TfR monoclonal antibody, TfRscFv-GAL4 fusion protein or mouse anti-GAL4 antibody used for detection of GAL4 domain, followed by FITC-labeled goat anti- mouse IgG. The proportion of FITC positive cell to the whole cell was calculated as showed in **A**, The statistical results presents there was significantly difference in the binding rate between the TfRscFv-GAL4 group and the negative control groups as showed in **B** (p<0.01), these results are representatives of four studies that have been done.

The binding of TfRscFv-GAL4 to the TfR was studied by flow cytometry using the TfR overexpression on tumor cell lines. We next sought to determine the antigen-binding activity for the TfRscFv-GAL4 fusion protein with various tumor cells. Flow cytometry analysis was employed for this purpose. It was noted that only the TfRscFv-GAL4 group (Figure 3A, TfRscFv-GAL4 group) and the positive controls (Figure 3A, Mouse anti-TfR group) showed positive results by flow cytometry assay, while the negative controls failed to detect any positive cells (Figure 3A, Mouse IgG group and GAL4 group). As shown in Figure 3B, the binding rate for TfRscFv-GAL4 fusion protein with 7 different tumor cells varied between 8.23% to 54.11%, and there was a significant difference in terms of the binding rate between the TfRscFv-GAL4 group and the negative control group (p<0.01). It showed that the purified TfRscFv-GAL4 retained its immunoreactivity comparable with that of the parental anti-TfR mAb. 

### Ag-binding potency of TfRscFv-GAL4 with various tissue microarray (TMA) sections

**Figure 4:**
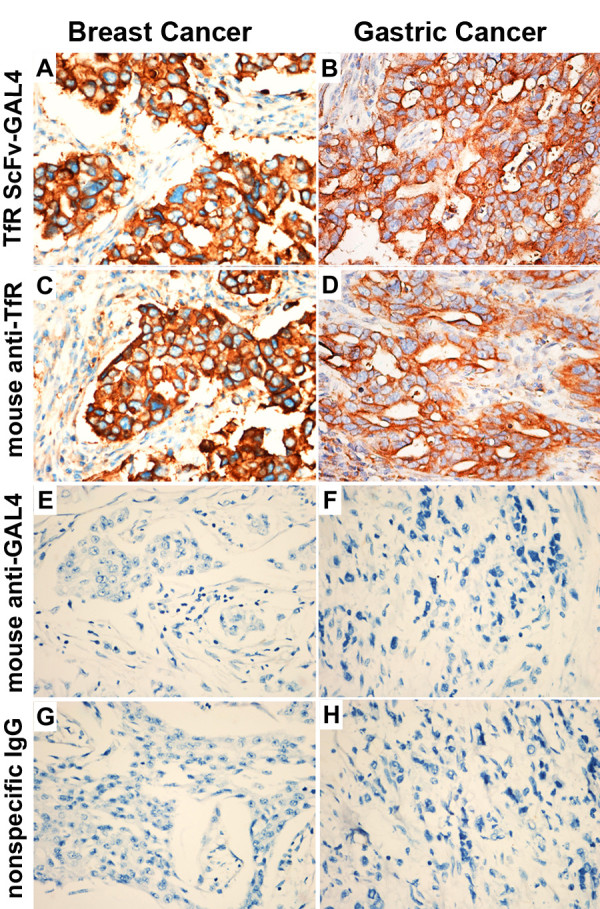
**Binding activity of TfRscFv-GAL4 fusion protein to gastric cancer and breast cancer in TMA. **140 gastric cancer tissue and 112 breast cancer tissue were embeded into TMA, dissect into 5μm and placed on one slide to make tissue microarray sections, TfRscFv-GAL4 single-chain antibodies were used to bind to the TMA sections (SP: × 400) **A**-**B**. Mouse anti-GAL4 antibody was used to bind the single-chain antibody, HRP conjugated goat anti-mouse antibody and DAB were used for detection of TfR protein expression. Parental mouse anti-TfR antibody was used as positive control (SP: × 400) **C**-**D**. The typical immunohistology results of positive binding activity with tumor tissues have been shown in **A**-**D** (SP: × 400). Both anti-GLA4 antibody and normal mouse IgG were used as negative control were typical immunohistology results of negative binding activity with tumor tissues (SP: × 400) **E-H**. Table 1 showed the statistic data of all tissue in immunohistology results.

According to the standard of TMA sections described in “Methods”, for gastric cancer TMA sections (total 140 cases), the available cases was 115 for TfRscFv-GAL4 fusion protein group, 95 for mouse anti-TfR antibody group, were 116 and 113 in mouse anti-GAL4 antibody group and mouse nonspecific IgG group, respectively. The available ratios were 85%, 70%, 85% and 83%, respectively. For gastric cancer TMA sections, the positive ratios were 75.32%, 78.46%, 0% and 0%, respectively (Figure 4A,C,E and G). For two breast cancer TMA sections (total 112 cases), the available cases were 110, 108, 109 and 108, respectively; the available ratios were 98%, 96%, 97% and 96%, respectively. For two breast cancer TMA sections, the positive ratios were 63.25%, 64.57%, 0% and 0%, respectively (Figure 4B,D,F, and H). The available ratios and positive ratios for TMA sections were showed in Table 1. There was a significant difference for the positive ratio between the TfRscFv-GAL4 group and the negative control group (mouse anti-GAL4 group and mouse nonspecific IgG group) (p<0.01). 

**Table 1:** The staining results and positive rate of the different tumor of TMA

	**Gastric cancer tissue microarray sections (n=140)**			**Breast cancer tissue microarray sections (n=112)**		
	**Available cases**	**Available ratio (%)**	**Positive ratio (%)**	**Available cases**	**Available ratio (%)**	**Positive ratio (%)**
TfRscFv-GAL4	115	85	75.32	110	98	63.25
anti-TfR mAb	95	70	78.46	108	96	64.57
anti-GAL4 mAb	116	85	0	109	97	0
nonspecific IgG	113	83	0	108	96	0

**Figure 5:**
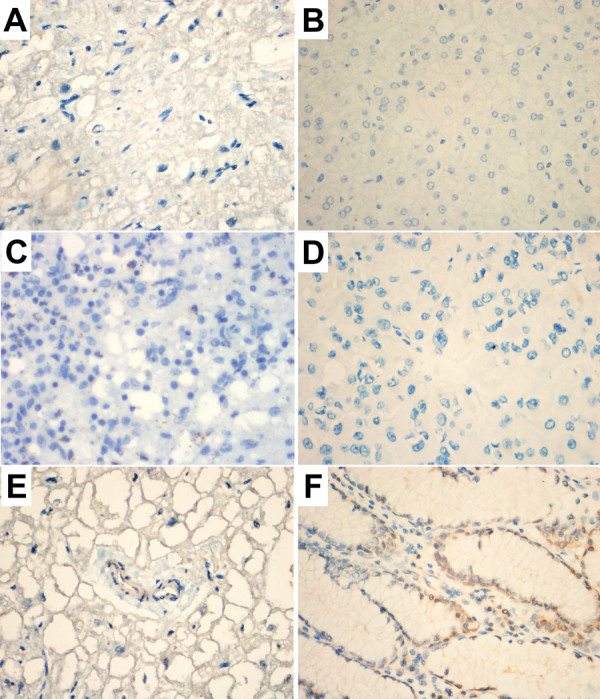
**Binding activity of TfRscFv-GAL4 fusion protein to normal tissues. **Normal tissue heart (**A**), liver (**B**), spleen (**C**), adrenal cortex (**D**), blood vessel (**E**) and stomach (**F**) were dissected to 5μm to creat the tissue slides, 10 individual samples were tested for each tissue. TfRscFv-GAL4 single-chain antibody was used for the detection of TfR expression, using parental mouse anti-TfR antibody as positive control. Typical results of each tissue were indicated in **A**-**F** (SP: × 400).

In contrast to the normal tissue sections, only 5 liver cases for the normal tissue sections showed weakly positive for the TfRscFv-GAL4 fusion protein group and mouse anti-TfR antibody group, while other tissues including the heart, spleen, adrenal cortex, blood vessels and stomach were negative for both groups (Figure 5A-F). Of note, all normal tissue sections were negative for the mouse anti-GAL4 group and mouse nonspecific IgG group. 

### Reactivity of TfRscFv-GAL4 with anti-GAL4 antibody

**Figure 6:**
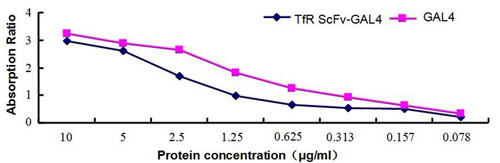
**Detection of GAL4 DNA binding domain in TfRscFv-GAL4 with ELISA. **TfRscFv-GAL4 was coated in the 96 well plates at different concentration, GAL4 protein was used a positive control. The absorption of OD495 nm was used for the detection of yellow products of OPD reacted by HRP.

To measure the binding activity of GAL4 domain for the purified TfRscFv-GAL4 fusion protein, we performed the ELISA analysis. The results revealed that both TfRscFv-GAL4 fusion protein and GAL4 protein bound to a rabbit anti-GAL4 antibody coated on the surface of a microtiter plate in a dose-dependent manner. Furthermore, the OD495 value increased with the parallel increase of the protein concentration (0.078~10 μg/ml) as shown in Figure 6. Thus, the GAL4 portion among TfRscFv-GAL4 fusion protein retained its high avidity for binding to rabbit anti-GAL4 antibody and it suggested the fusion protein re-naturation did not impair the GLA4 activity. 

### Functional characterization of the fusion proteins

**Figure 7:**
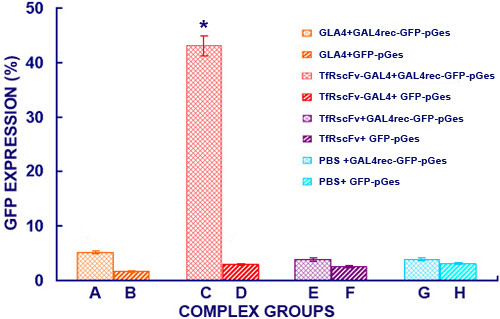
**Transfection efficiency of various complexes in HepG-2 cells determined by FCM Analysis. **First, 1 × 10^5^ cells in 6-welled plates were exposed to protein-DNA complex with molar ratio of 1:2.5 (protein to DNA) with or without fusion protein, and then they were further incubated for 48 h. For flow cytometry, HepG-2 cells after transfection with pCMV-GFP were washed, trypsinized and quantified using the FACS machine as described in Materials and Methods. Data are expressed as relative percentage of GFP-expression cells/total cells(mean±standard deviation, obtained from triplicate wells). Columns represented the transfection efficiency in different complex groups: (5.2±0.23)% for Column A, (1.7±0.18)% for Column B, (43.0±1.85)% for Column C, (2.9±0.23)% for Column D, (3.8±0.32)% for Column E, (2.5±0.15)% for Column F, (3.9±0.34)% for Column G, (3.1±0.26)% for Column H, The experiments were repeated 3 times with similar results. Column C showed 8~10 folds higher than other columns in transfection efficiency (^*^p<0.01).

The ability of protein-DNA binding was used to assess the biological activity of GLA4 in TfRscFv-GAL4 fusion protein. In the presence of the proper conjugating condition, the purified protein were then added to the plasmids with varying concentration to form the protein-DNA complex which were analyzed by electrophoresis on 4.5% gradient natural (non-SDS) PAGE followed by Western analysis, it showed that only one protein band could be seen but the moved protein band was dose-dependent with addition of DNA plasmids with the optimal molar ratio of protein to plasmid was 1:2.5 (data not shown). These constitutive proteins also served as a basis of comparison for the activities of the fusion protein. Flow cytometry was used to analyze the transfection efficiency of the protein-DNA complex mediated by TfRscFv portion. After a 48-hour incubation, expression of the GFP reporter gene was found in (43.0±1.85)% of HepG-2 cells that had been transfected with the mammalian expression plasmid of GAL4rec-GFP-pGes in the complex group (TfRscFv-GAL4+GAL4rec-GFP-pGes), in (5.2±0.23)% and (1.7±0.18)% of cells transfected with control and in (3.9±0.34)% transfected with naked DNA plasmid. It indicated that the endocytosis of the TfRscFv-targeted complex to tumor cells was significantly higher than those without TfRscFv as target molecule. In addition, Figure 7 in columns B,D,F, and G also illustrated that the capability of TfRscFv-GAL4 to mediate transfer GAL4rec-GFP-pGes expression plasmid into HepG2 cells as compared to GFP-pGes plasmid without GLA4, TfRscFv alone did not exhibit any DNA-binding capacity, confirming that the DNA-binding capacity of TfRscFv-GAL4 was strictly a function of the GAL4 component. 

## Discussion

Recently, non-viral vectors are under intense investigation as a safer alternative for gene therapy. For successful delivery, efficient and specific targeting of an active agent to the desired site is a critical factor for overcoming many barriers to carry DNA to nuclear localization and transcription [19]. Some of the most common non-viral vectors include polyethylenimine, dendrimers, chitosan, polylysine, and many types of peptides, which are generally cationic in nature and able to interact with plasmid DNA through electrostatic interactions [20]. Peptide-based vectors are advantageous over other non-viral strategies in that attachment of a peptide ligand to the polyplex will allow targeting to specific receptors and/or specific cell types, particularly in cancer cells [21]. However, an important drawback of the chemical coupling procedure is the difficulty in producing a reproducible and homogeneous product. Genetic engineering provides an alternative approach for large scale production of homogeneous Ab-GLA4 fusion proteins.

Previous studies have consistently suggested that TfR is expressed more abundantly in malignant tissues than that in their healthy counterparts [3-8]. Similarly, serum levels for soluble transferrin receptor (sTfR) in patients with hepatoma and haematologic malignancies are significantly increased as compared with those of normal controls [22]. Administration of anti-TfR antibody suppressed tumor cells growth *in vitro*[23]. Most importantly, TfR served as an endocytotic receptor which could carry the vector for uptake by the tumor cells [24]. Based on these findings, anti-TfR antibody is now considered as an alternative for diagnosis by imaging localization and for treatment targeting tumors [9,25-30]. The TfR-specific monoclonal or chimeric antibodies (mAbs) have been developed and used as an alternative therapeutic approach to kill malignant cells both *in vitro* and *in vivo*[31-33]. Although promising, this approach requires that unique chimeric molecules be constructed for each specific application or incapability to penetrate tissues for the high molecule weight of complete human-mouse chimeric TfR antibody. Thus, it also faces a formidable challenge which sometimes can lead to a decreased activity or loss of activity of one or both of the covalently conjugated partners. To overcome these limitations, it is therefore desirable to develop a universal delivery system that eliminates the need for a specific construct for each individual application.

The single chain antibody possesses the property to be integrated with different proteins for preparing some tumor targeting vectors (e.g. the complex constructed by chemical coupling method or the fusion protein constructed by gene engineering technique). Another critical feature for the single chain antibody is the low molecular weight (about 27 KDa, the 1/3 of chimeric antibody), which renders it with enhanced capability to penetrate tissues and cells. If TfRscFv is inserted into a eukaryocyte expression vector, the vector would be much more efficiently intaken by malignant cells. TfRscFv can be expressed in tumor cells by the vector. By using this approach, the efficiency for cancer therapy has been significantly improved and the dosage for anti-tumor drugs can also be reduced [34,35].

GAL4 is an 881-amino-acid protein with a Zn-Cys binuclear cluster type DNA-binding domain which is a positive regulator for galactose-induced gene expression [36]. The DNA-binding domain can specifically bind to a 17bp recognizing sequence motif (GAL4rec). Previous studies demonstrated that the fusion protein GAL4/Inv could be used in a DNA delivery system to target tumor cells [2]. Therefore, GAL4 could be used as a carrier for a plasmid containing the GAL4rec gene sequence.

In the current investigation, we generated and characterized bifunctionally active a TfRscFv-GAL4 fusion derived from plasmid pUC19 and pABgal4. TfRscFv-GAL4 with hexahistidine residues at the N-terminus were expressed in E. coli and recovered from the inclusion bodies with subsequent application of metal-chelate affinity chromatography. The fusion protein described in the present study is the first example of recombinant TfR single-chain monovalent antibodies with GAL4 DNA-binding activity produced in prokaryotic expression system, whereas either constituent component of recombinant protein showed individual activity through the following assays.

FCM assay revealed that the TfRscFv-GAL4 fusion protein is capable of binding to tumor cells, whose immune-reactivity is consistent with the parental anti-TfR monoclonal antibody. Therefore, our studies provided direct evidence that TfRscFv -GAL4 fusion protein, instead of GAL4 protein, could bind to various tumor cells. We also found that the binding rate for the TfRscFv-GAL4 fusion protein with seven different tumor cell lines varied between 8.23% and 54.11%. The differences for the binding rate may account for TfR being found expressed preferentially in the proliferation or differentiation stage of these cells rather than G0 stage, which was consistent with the results from Crepin R et al. [37] and our own results previously [12]. ELISA analysis indicated that the TfRscFv-GAL4 fusion protein can bind to anti-GAL4 antibody. Therefore, it showed that the fusion protein retained GLA4 activity and could also be served as a marker to evaluate the binding capability of TfRscFv with tumor cells. By using a mouse antibody against GAL4 and a FITC-labeled anti-mouse antibody, FCM assay was applied to detect the binding capability of the fusion protein with cells expressing TfR. Similarly, an HRP-labeled anti-mouse antibody could be used for detecting the binding capability of the fusion protein with tissues through TMA assay. Immunohistochemical studies in TMA indicated that both TfRscFv-GAL4 fusion protein and the mouse anti-TfR monoclonal antibody could bind to gastric cancer cells as well as breast cancer cells. In sharp contrast, neither GAL4 protein nor mouse non-specific IgG showed such binding activity, indicating that TfRscFv-GAL4 fusion protein binding to human gastric cancer cells and human breast cancer cells is through the TfRscFv domain but not GAL4 protein domain. Furthermore, except for 5 normal liver tissues with weekly binding activity, TfRscFv-GAL4 fusion protein and the mouse anti-TfR antibody failed to show any binding activity to normal tissues, suggesting that TfRscFv-GAL4 fusion protein was relatively specific to tumor tissues.

We designed the DNA-binding functional assay to further determine the fusion protein function. After conjugating the TfRscFv-GAL4 to the plasmid GAL4rec-pGes which was engineered to include a specific long sequence in the upstream activating sequence (5'-cggrnnrcynyncnccg-3', GAL4rec) and reporter gene GFP, protein-DNA complex incubated with hepatic carcinoma cells HepG2, and the GFP expression in cells was detected by fluorescence microscopy. Cells treated with complex TfRscFv-GAL4-GAL4rec-GFP-pGes showed a strong fluorescence signal, but cells treated with GAL4 lacking the TfRscFv, or those treated with TfRscFv without GLA4 portion, or naked DNA alone almost did not fluoresce. These data demonstrated that this process was in fact GLA4-DNA recognition and then mediated through the attachment of the scFv to the transferrin receptor on the tumor cells, and the fusion protein has the unique capacity to mediate gene transfer. Taken together, this protein-DNA may be considered as a promising candidate for developing novel tumor-targeted gene delivery for systemic gene therapy of various human cancers.

## Conclusions

We generated a novel DNA delivery vehicle containing single chain variable fragment of anti-transferrin receptor (TfRscFv) and GLA DNA binding domain (GAL4-DBD). GAL4-DBD is capable of binding DNA containing its specific binding sequence, and TfRscFv binding TfR on tumor cells is able to uptake by tumor cells through cell membranes to deliver DNA. This approach entailed covalently conjugating the fusion protein TfRscFv-GAL4 to the plasmid via a GAL4 at the 3’-end of the protein recognizing GAL4rec on the transfected plasmid (GAL4rec-GFP-pGes). Our results showed that this recombinant protein does not impair the immunological activity or targeting ability of the TfRscFv, as well as the functional avidity of GLA4 recognizing the GAL4rec sequence. The TfRscFv-GAL4 targeted the protein-DNA complex to tumor cells and enhanced the transfection efficiencies *in vitro*, but it did not exhibit the binding activity for normal tissues. The potential utility and appeal of this peptide-guided gene delivery lies with its internalization, the ability to control the nature of its constituent parts, and ease of generation. Briefly, this investigation provided basis for TfRscFv-GAL4 applications of orientation, tracing, imaging and targeted therapy on tumors.

## Methods

### Cells culture

All cancer cell lines such as HepG-2 (hepatocellular carcinoma cell line), HeLa (human cervical carcinoma cell line), MCF-7 (human breast cancer cell line), SLC-89 (human lung adenocarcinoma cell line), MDA-MB-231 (breast cancer cell line), HL-60 (Human promyelocytic leukemia cells) and MKN-28 (gastric cancer cell line) were purchased from ATCC. They were stored in our laboratory and cultured with the basal medium DMEM (GIBCO, USA) or RPMI 1640 (GIBCO, USA) containing 10% fetal bovine serum (GIBCO, USA) at 37°C with 5% CO_2_. For transfection, 1 × 10^5^ cells were initially seeded in 1 ml of medium in 12-well culture plates and transfected at 80% confluency.

### Prokaryotic expression vector of the TfRscFv-GAL4-pET28a

A fragment for the TfRscFv cDNA was directly amplified by PCR using the amplimers 5'-CGGCCATGGCCCACGTTCAGCTGCAGCAGT-3' and 5'-GGCGGAATTCTTTGATTTCCAGCTTGGTC-3' from a pUC19 plasmid containing the TfRscFv sequence as described before [12]. The fragment for GAL4 was released from a pABgal4 plasmid (kindly provided by Dr. Bert W. O' Malley, Baylor College of Medicine, Houston, TX) by digestions of *EcoRI* and *NotI* restriction endonucleases (Thermo Scientific Fermentas). The amplimers used were: 5'-CCG-GTATGGCTAGCCTGCAGAAGCTACTGTCTTCTATCG-3' and 5'-GAGCCGACCGGTACCTCAACTTACAGTCAACTGTC-3'. Both fragments for TfRscFv and GAL4 were introduced by ligation into expression vector pET28a (+) at the *NcoI* and *NotI* restriction site, respectively. In the resulting construct, pET28a (+), the hexahistidine sequence was fused in-frame to the GAL4 DNA-binding domain, followed by the (GGGGS)_3_ flexible peptide linker sequence and the carboxy-terminal 228 amino acids of TfRscFv (Figure 1). The resulted prokaryotic expression plasmid was referred as TfRscFv-GAL4-pET, which was subsequently transfected into *E*.*coli* BL_21_ (DE_3_) strain for plasmid DNA preparation. The inserted sequences were confirmed by restriction enzyme digestion with an agarose gel electrophoresis and DNA sequencing using an ABI PRISM ® 377 DNA Sequencer.

### Expression and purification of TfRscFv-GAL4 fusion protein

E. coli BL21 carrying plasmid TfRscFv-GAL4-pET was grown at an A_600_ of 0.5~0.7 overnight. An 0.5 ml aliquot of inoculate was added to 20 ml Luria-Bertani medium (LB) containing 25 μg/ml ampicillin and 50 mM glucose (LBG). The synthesis of recombinant TfRscFv-GAL4 fusion protein was induced with 1.0mM IPTG (isopropyl-β-D-thiogalacto-pyranoside) (Thermo Scientific Fermentas). Following 6h of incubation at different temperatures of 23°C, 30°C, 37°C, cells were harvested by centrifugation at 5000 *g* for 10 min at 4°C. Cell pellets were frozen at −20°C and resuspended in lysis buffer (6*M* guanidine-HCl, 20 m*M* sodium phosphate pH 7.8, 500 m*M* sodium chloride), sonicated 3×, and 0.45 μm filtered to remove insoluble debris. The extract of inclusion bodies was analyzed by 12.5% polyacrylamide gel electrophoresis (SDS-PAGE) under reducing conditions described as before [2,12]. Because TfRscFv-GAL4-pET expression vectors introduce a (His)_6_ tail at the C-terminus of the recombinant TfRscFv-GAL4, The solubilized fusion protein was purified by immobilized metal affinity chromatography (IMAC) through the binding of His-tag with Ni^2+^-nitrilotriacetate (Ni-NTA) agarose column (Invitrogen, USA) according to the manufacturer’s protocol as previously described [12,38] with minor modifications. After the solubilized inclusion bodies were passed through the column, the column was washed with 10 column volumes of 6M guanidine, 0.1 M Tris–HCl (pH 7.0) followed by 10 bed volumes of 6M urea, 50 mM Tris–HCl, 10 mM imidazole and 20 bed volumes of 6 M urea, 50 mM Tris–HCl, 50 mM imidazole. TfRscFv-GAL4 was eluted with 20 ml (4 bed volumes) of 6M urea, 50 mM Tris–HCl, 250 mM imidazole, and was then dialyzed in TEA (0.4 M L-arginine, 0.1 M Tris–HCl, 2 mM EDTA, pH 7.0) overnight. Optimal dilutions were determined empirically to avoid aggregation during refolding. Protein re-naturation was based on a procedure developed for the GLA4 fusion protein [39]. After dialysis, 60-75% of the re-natured protein was recovered in the soluble fraction after centrifugation. The concentration of fusion protein was calculated by bicinchoninic acid-based protein assay (BCA Protein Assay; Pierce, Rockford, IL), and plasmids pUC19, pABgal4 transformed E. coli BL21 to express protein as controls.

### Western blot analysis

*E*. *coli* BL_21_ lysates, and extracted inclusion bodies, which had been dialyzed in TEA before electrophoresis, or purified TfRscFv-GAL4, were resolved in 12% SDS–PAGE (Bio-Rad) under reducing conditions for Western blotting. Immunoblot analysis was carried out with the mouse mAb to GLA4 epitope (Sigma) as the primary antibody, peroxidase labeled horse anti-mouse IgG (Sigma) was used as the secondary antibody for GLA4 and results were visualized with DAB. Western blot with human TfR was used to determine the binding activity of purified TfRscFv-GAL4. Human TfR was electrophoresed in SDS-PAGE and blotted as described above. The membrane was sequentially incubated with the TfRscFv-GAL4 (primary antibody), the GLA4 mAb, biotinylated horse anti-mouse IgG (secondary antibody).

### Flow cytometry

The antigen-binding activity of TfRscFv in TfRscFv-GAL4 fusion protein was analysed by an indirect Immunofluorescence Assay as described before [12,38]. Briefly, 2 × 10^5^ of HepG-2, HeLa, MCF-7, HL-60, MDA-MB-231, MKN28 and SLC-89 cells were collected at logarithmic phase. After washed with washing buffer (cold PBS, 1% BSA, 1mM CaCl_2_, 1mM MgCl_2_), the cells were incubated with TfRscFv-GAL4 for 20 min at 4°C, then treated with a mouse anti-GAL4 antibody and stained with FITC goat anti-mouse IgG. The cells were next analyzed by flow cytometry using the Cell Quest software package (Becton–Dickinson FACScalibur, USA). A mouse anti-TfR monoclonal antibody was used as a positive control, while the GAL4 protein and mouse non-specific IgG were used for isotype controls.

### Construction of tissue microarrays (TMA) and Immunohistochemistry (IHC)

Paraffin-embedded tissue samples based on availability of resected tissues from 140 patients with gastric cancer and 112 patients with breast cancer which were obtained from the archives of Department of Pathology, Medical School of Jianghan University, Tongji Hospital of HUST and Department of Pathology, Medical School of Peking University. Tissue sections originated from autopsy samples such as from heart, blood vessels, liver, adrenal cortex, spleen and stomach were used as normal controls (each tissue included 5 cases), which were provided by the Department of Pathology, Medical School of Jianghan University and Tongji Hospital of HUST. The formalin-fixed, paraffin-embedded tissue blocks and the corresponding histological HE-stained slides were overlaid for tissue TMA sampling. Representative areas of tumor tissues were marked on the slides. A tissue arraying instrument (Beecher Instruments, Silver Spring, MD, USA) was used to punch triplicate 0.6-mm-diameter cylinders of tissue from selected cancer areas of individual donor tissue block and re-emb into a recipient paraffin block at a predefined position. Subsequently, multiple sections (5μm thick) were cut from the TMA tissue array.

IHC was employed to measure the Ag-binding rates for TfRscFv-GAL4 fusion protein with various tumor TMA sections. The immunohistochemical study of Ag-binding was performed using a standard streptavidin-peroxidase method described previously [40]. The endogenous peroxidase activity was blocked with 3% H_2_O_2_ for 10 minutes. For antigen retrieval, slides were immersed in 10 mM citrate buffer (pH 6.0) and boiled for 15 minutes in a microwave oven. Non-specific binding was blocked by 5% normal goat serum for 10 minutes. The slides were treated with a mouse anti-GAL4 antibody after incubated with TfRscFv-GAL4 against TfR expressing on TMA at 4°C overnight in a moist chamber followed by incubating with HRP labeled goat anti-mouse IgG (Southern Biotechnology Associates, USA) for 20 mins at 37°C, and then with streptavidin-peroxidase conjugate, each for 30 mins at room temperature. The parental mouse anti-TfR antibody (Mouse anti-TfR group) was used as the positive control, and Mouse IgG group and GAL4 group, was used as the negative control, respectively. The TMA were finally stained with 3, 5-diaminobenzidine (DAB) reagents and incubated with hematoxylin to stain the nuclei, and results were evaluated under a light microscope. For the evaluation of TMA staining, a semi-quantitative scoring criterion was used, in which both staining intensity and positive cells percentage were scored. A staining index (with values from 0 to 12) was obtained as the intensity of TfR staining (0=negative, 1=weakly positive, 2=positive, 3=strongly positive) times the proportion of immune-positive tumor cells (0%=0; <10%=1; 10%≤ to<50%=2; 50%≤to <75%=3; ≥75%=4; both cross products ≤1 was judged as negative value and ≥2 as positive value. All histological evaluations were carried out in a double-blind manner by two expert pathologists (DH and YQ).

### ELISA-based binding assay

It was employed to an ELISA-based assay to determine the protein activity of GAL4 domain for the TfRscFv-GAL4 fusion protein when bound and not bound to its special anti-GLA4 antibody. Briefly, the GAL4 or TfRscFv-GAL4 was coated in a 96-well plate (100 μl/well) at 4°C overnight. After washes with PBS (containing 0.005% Tween-20), the plate was blocked for 2 h at 37°C, followed by incubation with a rabbit anti-GAL4 antibody (0.1 μg/ml, Invitrogen, USA) for 2 h at 37°C. Next, HRP labeled goat anti-rabbit IgG (1:5000, 100 μl/well, Southern Biotechnology Associates, USA) was applied and incubated for 1h at 37°C. Freshly prepared substrate solution (100 μl/well) was finally added and incubated for 15 min at room temperature, and the results were read by a photometer at 495 nm (TECAN, USA) after application of stop solution (50 μl/well) for color development. As a negative control, the conventional TfRscFv was used.

### Functional assay of GAL4-DNA binding

The protein-DNA complex was generated prior to GAL4-DNA binding assay. Mammalian expression vectors GAL4rec-GFP-pGes (Shanghai BlueGene Biotech Co., Ltd. Shanghai, China) encoding the green fluorescent protein (GFP) with the human cytomegalovirus promoter (pCMV), was made by ligating the consensus 17-bp sequence(5'-cggrnnrcynyncnccg-3') recognizing GAL4, The resulting plasmids, which contained eight tandem copies of the target oligonucleotide and its sequence was identified by our research group previously(unpublished data), amplified in the DH5 a strain of *E*. *coli*, and purified according to E.Z.N.A.®Plasmid Maxiprep Kit (OMEGA, USA) and their concentrations were measured by UV meter (Gene Spec I, Japan).

Dilutions of purified and re-natured TfRscFv-GAL4 in re-naturation buffer were mixed with Plasmid DNA (GAL4rec-GFP-pGes plasmid or GFP-pGes plasmid) in a total volume of 20 μl with varying concentration ratio of protein-DNA between 0.5 to 10.0. The mixtures were conjugated in the reaction system of protein-DNA linking buffer (25 mM HEPES pH 7.6, 50 mM KCL, 5 mM MgCL_2_, 100 μM ZnCl_2_, 0.1 mM EDTA, 0.1% Nonidet P40, 10% glycerol, 200 μg/ml BSA, 50μl/ml poly(dI-dC)) for 15 min at 25°C. The solution was mixed well for 10 min by gently inversion several times to produce the complex of TfRscFv-GAL4 and GAL4rec-GFP-pGes. Then, 4.5% gradient (non-SDS) PAGE followed by Western analysis was also used to assess the optimal molar ratio of protein to plasmid as 1:2.5. In Accordance with the complex transfection protocol [1], the hepatic cancer cells HepG-2 (5x10^5^/well) were seeded in 12-well culture plates 24h before transfection. The culture medium was replaced with free-FBS RPMI-1640 medium before the sequential addition of 0.1 ml protein-DNA complexes of TfRscFv-GAL4 and GAL4rec-GFP-pGes, after incubation for 5h in 5% CO_2_ at 37°C, 1 ml of medium with 10% FBS was added and incubated for another 48h. Alternatively, the purified protein GAL4, TfRscFv, or the vector (GFP-pGes) complex as different controls and phosphate buffered saline(PBS) as a blank control, respectively. On the day of harvest, cells transfected with pCMV-GFP were washed with PBS and trypsinized. The transfection efficiency was evaluated by scoring the percentage of cells expressing GFP using a FACS Calibur System (Becton-Dickinson). The experiments were performed in triplicate, and 10,000 cells were counted in each experiment.

### Statistical analysis

All results from each experiment were expressed as mean ± SEM with number (n) of observations. Sets of data were compared with an analysis of variance (ANOVA) or a Student’s *t* test. Differences were considered statistically significant when P < 0.01. Symbols used in figures were (*) for P > 0.05 and NS for no significant difference. All statistical tests were performed using GraphPad Prism version 4.0 for Windows (Graph pad Software).

### Ethics statement

The materials and methods of our study were approved by the Ethics Committee of Tongji Hospital of Huazhong University of Science and Technology (HUST), Jianghan University and Peking University. The written informed consent was obtained from each participating patient (or patient guardian) before entry into the study. The study was conducted in accordance with the *Declaration of Helsinki*.

## Competing interests

The authors declare that they have no competing interests.

## Authors’ contributions

ZW, QY and HH designed and carried out the study and drafted the manuscript. TL, JL, SZ helped to draft the manuscript. XZ performed the the statistical analysis. ZH, CW, ZY and GS participated in its design and coordination and helped to draft the manuscript. All authors read and approved the final manuscript.

## References

[B1] Kim ES Yang SW Hong DK Kim WT Kim HG Lee SK Cell-penetrating DNA-binding protein as a safe and efficient naked DNA delivery carrier *in vitro* and *in vivo* Biochem Biophys Res Commun 2010 392 1 9 15 10.1016/j.bbrc.2009.12.135 20043881

[B2] Paul RW Weisser KE Loomis A Sloane DL LaFoe D Atkinson EM Overell RW Gene transfer using a novel fusion protein, GAL4/invasin Hum Gene Ther 1997 8 10 1253 1262 10.1089/hum.1997.8.10-1253 9215742

[B3] Daniels TR Delgado T Rodriguez JA Helguera G Penichet ML The transferrin receptor part I: Biology and targeting with cytotoxic antibodies for the treatment of cancer Clin Immunol 2006 121 2 144 158 10.1016/j.clim.2006.06.010 16904380

[B4] Kohgo Y Kondo H Mogi Y Niitsu Y Mechanism and clinical significance of soluble hepatic cell-surface receptors Targeted Diagn Ther 1991 4 305 319 1797162

[B5] Kollia P Samara M Stamatopoulos K Belessi C Stavroyianni N Tsompanakou A Athanasiadou A Vamvakopoulos N Laoutaris N Anagnostopoulos A Molecular evidence for transferrin receptor 2 expression in all FAB subtypes of acute myeloid leukemia Leuk Res 2003 27 12 1101 1103 10.1016/S0145-2126(03)00100-0 12921947

[B6] Qing Y Shuo W Zhihua W Huifen Z Ping L Lijiang L Xiaorong Z Liming C Daiwen X Yu H The *in vitro* antitumor effect and *in vivo* tumor-specificity distribution of human-mouse chimeric antibody against transferrin receptor Cancer Immunol Immunother 2006 55 9 1111 1121 10.1007/s00262-005-0105-7 16341531 PMC11030686

[B7] Widera A Norouziyan F Shen WC Mechanisms of TfR-mediated transcytosis and sorting in epithelial cells and applications toward drug delivery Adv Drug Deliv Rev 2003 55 11 1439 1466 10.1016/j.addr.2003.07.004 14597140

[B8] Yang DC Wang F Elliott RL Head JF Expression of transferrin receptor and ferritin H-chain mRNA are associated with clinical and histopathological prognostic indicators in breast cancer Anticancer Res 2001 21 1B 541 549 11299801

[B9] Daniels TR Delgado T Helguera G Penichet ML The transferrin receptor part II: targeted delivery of therapeutic agents into cancer cells Clin Immunol 2006 121 2 159 176 10.1016/j.clim.2006.06.006 16920030

[B10] Habashy HO Powe DG Staka CM Rakha EA Ball G Green AR Aleskandarany M Paish EC Douglas Macmillan R Nicholson RI Transferrin receptor (CD71) is a marker of poor prognosis in breast cancer and can predict response to tamoxifen Breast Cancer Res Treat 2010 119 2 283 293 10.1007/s10549-009-0345-x 19238537

[B11] Huang RK Steinmetz NF Fu CY Manchester M Johnson JE Transferrin-mediated targeting of bacteriophage HK97 nanoparticles into tumor cells Nanomedicine (Lond) 2011 6 1 55 68 10.2217/nnm.10.99 21182418 PMC3091364

[B12] Liu J Xiao D Zhou X Wen X Dai H Wang Z Shen X Dai W Yang D Shen G Preparation and identification of scFv and bsFv against transferrin receptor J Huazhong Univ Sci Technolog Med Sci 2008 28 6 621 625 10.1007/s11596-008-0601-z 19107352

[B13] Peng JL Wu S Zhao XP Wang M Li WH Shen X Liu J Lei P Zhu HF Shen GX Downregulation of transferrin receptor surface expression by intracellular antibody Biochem Biophys Res Commun 2007 354 4 864 871 10.1016/j.bbrc.2007.01.052 17266924

[B14] Shen X Hu GB Jiang SJ He FR Xing W Li L Yang J Zhu HF Lei P Shen GX Engineering and characterization of a baculovirus-expressed mouse/human chimeric antibody against transferrin receptor Protein Eng Des Sel 2009 22 12 723 731 10.1093/protein/gzp054 19825853

[B15] Liu Y Tao J Li Y Yang J Yu Y Wang M Xu X Huang C Huang W Dong J Targeting hypoxia-inducible factor-1alpha with Tf-PEI-shRNA complex via transferrin receptor-mediated endocytosis inhibits melanoma growth Mol Ther 2009 17 2 269 277 10.1038/mt.2008.266 19066596 PMC2835063

[B16] Giniger E Varnum SM Ptashne M Specific DNA binding of GAL4, a positive regulatory protein of yeast Cell 1985 40 4 767 774 10.1016/0092-8674(85)90336-8 3886158

[B17] Fominaya J Wels W Target cell-specific DNA transfer mediated by a chimeric multidomain protein. Novel non-viral gene delivery system J Biol Chem 1996 271 18 10560 10568 10.1074/jbc.271.18.10560 8631856

[B18] Silver PA Keegan LP Ptashne M Amino terminus of the yeast GAL4 gene product is sufficient for nuclear localization Proc Natl Acad Sci U S A 1984 81 19 5951 5955 10.1073/pnas.81.19.5951 6091123 PMC391836

[B19] Martin ME Rice KG Peptide-guided gene delivery AAPS J 2007 9 1 E18 E29 10.1208/aapsj0901003 17408236 PMC2751301

[B20] Mahato RI Non-viral peptide-based approaches to gene delivery J Drug Target 1999 7 4 249 268 10.3109/10611869909085509 10682905

[B21] Deshayes S Morris MC Divita G Heitz F Cell-penetrating peptides: tools for intracellular delivery of therapeutics Cell Mol Life Sci 2005 62 16 1839 1849 10.1007/s00018-005-5109-0 15968462 PMC11139131

[B22] Takubo T Kumura T Nakao T Nakamae H Aoyama Y Nishiki S Kinoshita Y Koh KR Ohta K Yamane T Clinical usefulness of combined measurements of serum soluble transferrin receptor levels and serum interleukin-18 levels at determination of serum KL-6 levels in haematologic malignancies Acta Haematol 2000 104 2–3 141 143 11154993 10.1159/000039750

[B23] Hong Y Yang J Shen X Zhu H Sun X Wen X Bian J Hu H Yuan L Tao J Lei P Shen G Sinomenine hydrochloride enhancement of the inhibitory effects of anti-transferrin receptor antibody dependent on the COX-2 pathway in human hepatoma cells Cancer Immunol Immunother 2012 Epub ahead of print 10.1007/s00262-012-1337-y PMC11028739 22941037

[B24] Vijaykumar V Topp EM Diffusion of an anti-transferrin receptor antibody in cultured murine melanoma cell layers Pharm Res 1995 12 12 1907 1916 10.1023/A:1016424321378 8786965

[B25] Asai T Trinh R Ng PP Penichet ML Wims LA Morrison SL A human biotin acceptor domain allows site-specific conjugation of an enzyme to an antibody-avidin fusion protein for targeted drug delivery Biomol Eng 2005 21 6 145 155 10.1016/j.bioeng.2004.10.001 15748688

[B26] Hogemann-Savellano D Bos E Blondet C Sato F Abe T Josephson L Weissleder R Gaudet J Sgroi D Peters PJ The transferrin receptor: a potential molecular imaging marker for human cancer Neoplasia 2003 5 6 495 506 14965443 10.1016/s1476-5586(03)80034-9 PMC1502574

[B27] Hsu CP Ko JL Shai SE Lee LW Modulation of telomere shelterin by TRF1 [corrected] and TRF2 interacts with telomerase to maintain the telomere length in non-small cell lung cancer Lung Cancer 2007 58 3 310 316 10.1016/j.lungcan.2007.06.019 17681636

[B28] Pirollo KF Dagata J Wang P Freedman M Vladar A Fricke S Ileva L Zhou Q Chang EH A tumor-targeted nanodelivery system to improve early MRI detection of cancer Mol Imaging 2006 5 1 41 52 16779969

[B29] Rodriguez JA Helguera G Daniels TR Neacato II Lopez-Valdes HE Charles AC Penichet ML Binding specificity and internalization properties of an antibody-avidin fusion protein targeting the human transferrin receptor J Control Release 2007 124 1–2 35 42 17884229 10.1016/j.jconrel.2007.08.020

[B30] Shinohara H Fan D Ozawa S Yano S Van Arsdell M Viner JL Beers R Pastan I Fidler IJ Site-specific expression of transferrin receptor by human colon cancer cells directly correlates with eradication by antitransferrin recombinant immunotoxin Int J Oncol 2000 17 4 643 651 10995873 10.3892/ijo.17.4.643

[B31] Callens C Moura IC Lepelletier Y Coulon S Renand A Dussiot M Ghez D Benhamou M Monteiro RC Bazarbachi A Recent advances in adult T-cell leukemia therapy: focus on a new anti-transferrin receptor monoclonal antibody Leukemia 2008 22 1 42 48 10.1038/sj.leu.2404958 17898788

[B32] Shen X Zhu HF He FR Xing W Li L Liu J Yang J Pan XF Lei P Wang ZH An anti-transferrin receptor antibody enhanced the growth inhibitory effects of chemotherapeutic drugs on human non-hematopoietic tumor cells Int Immunopharmacol 2008 8 13–14 1813 1820 18817895 10.1016/j.intimp.2008.08.022

[B33] Trowbridge IS Lopez F Monoclonal antibody to transferrin receptor blocks transferrin binding and inhibits human tumor cell growth in vitro Proc Natl Acad Sci U S A 1982 79 4 1175 1179 10.1073/pnas.79.4.1175 6280171 PMC345924

[B34] Pastan I FitzGerald D Recombinant toxins for cancer treatment Science 1991 254 5035 1173 1177 10.1126/science.1683495 1683495

[B35] Xu L Huang CC Huang W Tang WH Rait A Yin YZ Cruz I Xiang LM Pirollo KF Chang EH Systemic tumor-targeted gene delivery by anti-transferrin receptor scFv-immunoliposomes Mol Cancer Ther 2002 1 5 337 346 12489850

[B36] Traven A Jelicic B Sopta M Yeast Gal4: a transcriptional paradigm revisited EMBO Rep 2006 7 5 496 499 10.1038/sj.embor.7400679 16670683 PMC1479557

[B37] Crepin R Goenaga AL Jullienne B Bougherara H Legay C Benihoud K Marks JD Poul MA Development of human single-chain antibodies to the transferrin receptor that effectively antagonize the growth of leukemias and lymphomas Cancer Res 2010 70 13 5497 5506 10.1158/0008-5472.CAN-10-0938 20530676

[B38] Li JY Sugimura K Boado RJ Lee HJ Zhang C Duebel S Pardridge WM Genetically engineered brain drug delivery vectors: cloning, expression and *in vivo* application of an anti-transferrin receptor single chain antibody-streptavidin fusion gene and protein Protein Eng 1999 12 9 787 796 10.1093/protein/12.9.787 10506289

[B39] Reece RJ Rickles RJ Ptashne M Overproduction and single-step purification of GAL4 fusion proteins from Escherichia coli Gene 1993 126 1 105 107 10.1016/0378-1119(93)90596-U 8472950

[B40] He WP Zhou J Cai MY Xiao XS Liao YJ Kung HF Guan XY Xie D Yang GF CHD1L Protein is overexpressed in human ovarian carcinomas and is a novel predictive biomarker for patients survival BMC Cancer 2012 12 1 437 10.1186/1471-2407-12-437 23020525 PMC3551745

